# Surface Defect Detection of Fresh-Cut Cauliflowers Based on Convolutional Neural Network with Transfer Learning

**DOI:** 10.3390/foods11182915

**Published:** 2022-09-19

**Authors:** Yaodi Li, Jianxin Xue, Kai Wang, Mingyue Zhang, Zezhen Li

**Affiliations:** 1 College of Agricultural Engineering, Shanxi Agricultural University, Jinzhong 030801, China; 2 College of Food Science and Engineering, Shanxi Agricultural University, Jinzhong 030801, China

**Keywords:** fresh-cut cauliflower, surface defect detection, convolutional neural network, transfer learning, classification

## Abstract

A fresh-cut cauliflower surface defect detection and classification model based on a convolutional neural network with transfer learning is proposed to address the low efficiency of the traditional manual detection of fresh-cut cauliflower surface defects. Four thousand, seven hundred and ninety images of fresh-cut cauliflower were collected in four categories including healthy, diseased, browning, and mildewed. In this study, the pre-trained MobileNet model was fine-tuned to improve training speed and accuracy. The model optimization was achieved by selecting the optimal combination of training hyper-parameters and adjusting the different number of frozen layers; the parameters downloaded from ImageNet were optimally integrated with the parameters trained on our own model. A comparison of test results was presented by combining VGG19, InceptionV3, and NASNetMobile. Experimental results showed that the MobileNet model’s loss value was 0.033, its accuracy was 99.27%, and the F1 score was 99.24% on the test set when the learning rate was set as 0.001, dropout was set as 0.5, and the frozen layer was set as 80. This model had better capability and stronger robustness and was more suitable for the surface defect detection of fresh-cut cauliflower when compared with other models, and the experiment’s results demonstrated the method’s feasibility.

## 1. Introduction

Cauliflower (*Brassica oleracea* L. botrytis) is a variety of Brassica wild cabbage. It has become a popular vegetable in the world because of its rich nutritional value, high yield, and large economic benefits.

Fresh-cut fruits and vegetables, also known as semi-processed fruits and vegetables, refer to fresh fruits and vegetables as raw materials, after a series of processing activities such as grading, cleaning, trimming, preserving, packaging, etc., and then after low-temperature transportation into the freezer sales of ready-to-eat or ready-to-use fruit and vegetable products. Fresh-cut cauliflower not only maintains the original freshness of the cauliflower itself but processing also makes it clean and hygienic, which can meet the needs of people’s contemporary pursuit of a natural, nutritious, fast-paced lifestyle and other aspects. Therefore, the quality grading of fresh-cut cauliflower is an important part of its processing. At present, the surface defect detection of fresh-cut cauliflower is largely performed manually, which is slow and has low precision. Under such detection conditions, it is hard to effectively guarantee the quality of fresh-cut cauliflower, which largely restricts the scale of its production, transportation, and storage in the fresh-cut cauliflower industry. Thus, recognizing and detecting fresh-cut cauliflower surface defects accurately is necessary and urgent. This solution will be of significant economic benefit and have a significant future planning meaning for the development of the fresh-cut cauliflower industry.

## 2. Related Work

With the continuous innovation of modern science and technology, machine vision technology has been extensively used for the surface defect detection of agricultural products. Based on computer vision technology, J. Blasco et al. adopted a region-oriented segmentation algorithm for citrus fruits’ external defect detection and achieved a 95% detection accuracy rate [1]. Li Jinwei et al. proposed a fast gray cut-off segmentation method that separated suspected defects from potato surfaces and a ten-color model for potato surface defect recognition, which enabled defect detection with 95.7% accuracy [2]. Zhao Juan et al. designed a system to capture all aspects of apple surface information and achieved a 92.5% correct recognition rate in the identification of external apple defects [3].

With the accelerated development of artificial intelligence and the application of state-of-the-art deep learning technology in numerous domains [4,5,6,7,8,9,10], traditional machine learning methods have been gradually replaced. Traditional machine learning relies on manually designed feature extractors, and it is difficult to achieve complete extraction of image information, so it has been difficult to make a significant breakthrough. The convolutional neural network (CNN) has shown significant advantages in the field of image recognition due to its ability to automatically acquire the deep characteristics of the original image and is widely used in the quality inspection of agricultural products [11,12], especially in the disease detection [13,14,15], defect detection [16,17,18], maturity detection [19,20,21], and grading [22,23,24] of agricultural products. Xue Zhao et al. developed a DTL-SE-ResNet50 vegetable disease recognition model based on a CNN to identify tomato powdery mildew, leaf mold, and cucumber downy mildew in simple and complex contexts [25]. The model was also compared with EfficientNet, AlexNet, VGG19, and InceptionV3 models, and the recognition accuracy of DTL-SE-ResNet50 reached 97.24% under the same experimental conditions, which outperformed the other four models. Guoyang Zhao et al. designed and developed a deep learning-based full surface recognition sorting system for soybean seeds with the research goal of accurately sorting high-quality soybean seeds [26]. The sorting system collected all feature information of a seed’s surface by an alternate rotation mechanism and accurately classified seeds by using the deep learning model, with a 98.87% sorting accuracy rate and 222 seeds/min sorting speed. Long Jiehua et al. proposed an improved Mask R-CNN method for segmenting tomato fruits with different ripeness levels under a greenhouse environment [27]; the mean average precision of the improved Mask R-CNN model was 95.45% for segmenting tomatoes at green ripe, half ripe, and ripe stages. The improved Mask R-CNN model was also deployed to a picking robot and then tested in the field, and the model achieved a 90% correct recognition rate. Zhiheng Lu et al. designed a machine vision-based automatic winter jujube grading robot and proposed an image processing method combining YOLOv3 algorithm with manual features for calculating the ripeness of winter jujubes [28]. Through experiments, the algorithm achieved 97.28% accuracy in grading the maturity of winter jujube, and the detection time was 1.39 s for each date.

Deep learning has been widely used in agricultural product quality detection, and a CNN is a typical representative of deep learning. Various research results have shown that a CNN not only has a strong learning ability but also can realize the automatic extraction of image features and achieve a good recognition effect. However, reports about the detection of fresh-cut cauliflower surface defects are rare. To achieve automatic detection of fresh-cut cauliflower surface defects, this study combined a CNN with the transfer learning method and established a fresh-cut cauliflower surface defect detection model. This study has referential meaning for the detection of surface defects in fresh-cut cauliflower and broadens the ideas for quality inspection in the field of fresh-cut fruits and vegetables.

## 3. Materials and Methods

### 3.1. Dataset Acquisition

**Figure 1:**
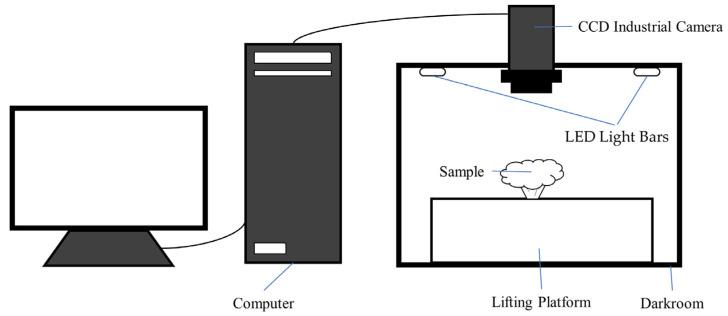
The image acquisition facilities.

In this experiment, “Xuebai” cauliflower was taken as the research subject. “Xuebai” cauliflower is a variety of cauliflower in China with uniform, firm flower bulbs and good merchantability. The image acquisition facilities included a computer, darkroom, lifting platform, CCD industrial camera, and LED light bars. The background of the cauliflower image is white, which is convenient to reduce the interference of other environmental factors in the image acquisition. The shooting angle of the camera was vertically downward, 10 cm from the cauliflower. The image acquisition facilities are shown in Figure 1. Fresh-cut cauliflower samples were placed by the experimenter in the center of the lifting platform in the darkroom and then photographed by a computer-controlled CCD industrial camera.

Fresh-cut cauliflower samples can be classified into four categories: healthy, diseased, browning, and mildewed. Healthy cauliflowers had full flower bulbs and snowy white color. Diseased cauliflowers were due to bacterial infection of stems, resulting in local blackening of stems, and had lost edible value. The specific performance of browning cauliflowers was that the surface of the cauliflower had brown spots and rot symptoms. Mildewed cauliflowers were infected by gray mold disease, resulting in gray–white colonies on the surface. For data collection, 479 cauliflower samples were chosen. Image enhancement techniques were used to increase the number of datasets so that the model obtained enough information during training to improve its generalization ability [29].

**Figure 2:**
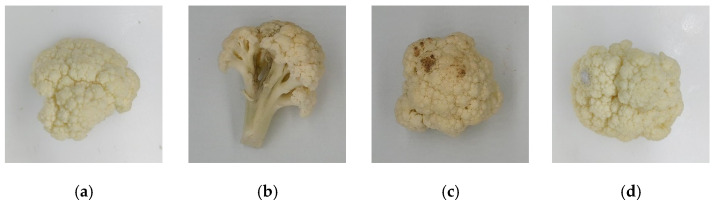
Four categories of fresh-cut cauliflower samples: (**a**) healthy; (**b**) diseased; (**c**) browning; (**d**) mildewed.

The following image enhancement techniques were used: translation, inversion, rotation, saturation, enhancement, and brightness adjustment. Finally, 4790 images were acquired. The dataset descriptions of the original dataset, augmented dataset, training set, validation set, and test set are shown in Table 1. Part of the original images is provided in Figure 2.

This research used Windows 11 operating system, AMD R7-5800H, CPU@2.9GHz, 16GB RAM, 500GB Hard-Disk drive, and 8GB NVIDIA RTX 3070 GPU. The algorithm adopted CUDA11.3.1 and CUDNN8.2.1 libraries. Python version was 3.7.3. TensorFlow version was 2.5. All programs were run on PyCharm.

### 3.2. CNN and Transfer Learning

CNN is a variety of multi-layer perceptrons, and is often applied to image classification in the field of deep learning. The training of the CNN model usually needs tremendous data and calculation, and it is difficult to achieve the desired effect if the dataset of training samples is relatively small. Training from scratch can be time-consuming and has a high demand for computer hardware. To solve these problems, we used the method of transfer learning [30], by using the similarity between data or tasks, migration of existing trained models to new models to help train new models, and improving the model according to assigned tasks, such as fine-tuning strategy. The main idea was to adjust one or more layers of the pre-training model to obtain better training results.

### 3.3. Model Establishment

**Figure 3:**
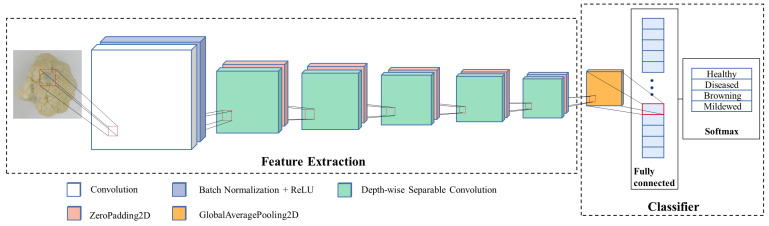
Architecture of MobileNet.

Currently, the mainstream pre-training models are VGGxNet [31], ResNet50 [32], Xception [33], InceptionV3 [34], and MobileNet [35]. However, some models have vast quantities of parameters, such as VGG16, which has 138,357,544 parameters, resulting in a model size of 528MB. Models that are too large require high computing power and large memory size of hardware devices for training and running, this makes it a hard issue for the model to function properly on mobile devices as well as embedded devices. Based on the above-mentioned issues, we need to construct a lightweight model to meet the needs of detecting surface defects of fresh-cut cauliflower. MobileNet, proposed by the Google team in 2017, is a lightweight CNN focused on mobile terminals and embedded devices. Compared with traditional CNN, it greatly reduces the model parameters and operations with a small reduction in accuracy. On ImageNet classification, MobileNet has only 0.9% less accuracy compared to VGG16, while being 32 times smaller, the accuracy of MobileNet is 0.8% higher than GoogleNet and is 1.6 times smaller. Through comprehensive analysis, MobileNet, with a small size and good recognition performance, was selected for this experiment. The architecture of MobileNet is shown in Figure 3.

**Figure 4:**
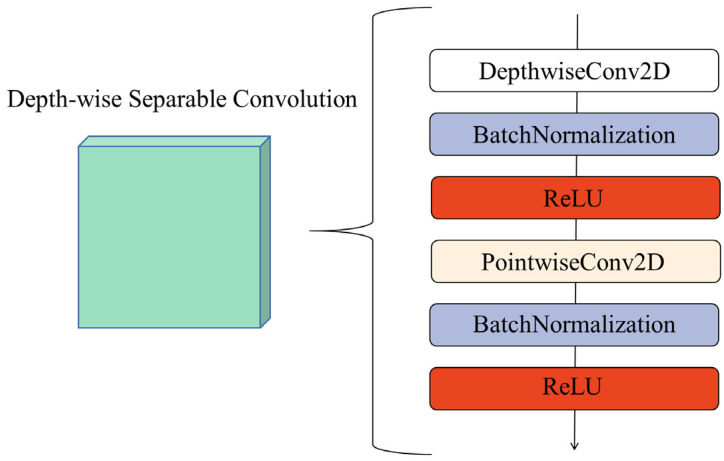
The structure of depth-wise separable convolution.

The architecture of MobileNet includes 13 depth-wise separable convolutions, and the structure of a depth-wise separable convolution is shown in Figure 4. Each DSC contains a DepthwiseConv2D and a PointwiseConv2D, and each Conv2D is followed by a Batch Normalization [36] and a ReLU.

For feature extraction of different defects on the fresh-cut cauliflower surface, we first imported the parameters from ImageNet, then removed the fully connected layer and SoftMax layer, and kept only the convolutional module for extracting image features, which was used as the feature extraction module of the deep transfer learning model in this research.

For the classification of different defects on the fresh-cut cauliflower surface, the number of output categories in the last layer of MobileNet was changed from 1000 to 4, and a dropout layer was added after the global average pooling layer to suppress the overfitting of the model [37]. Finally, the results were output with a softmax layer. These were used as the classifier module of the deep transfer learning model in this research.

## 4. Training and Evaluation

### 4.1. CNN Training

This research conducted training towards the developed MobileNet network. In order to obtain better model training performance, we utilized GPU to accelerate the training of the model [38]. To ensure that the model learned the data features sufficiently during training, the number of training epochs was configured as 100 in this research. For the sake of maintaining a balance between memory capacity and memory efficiency, the batch size for each training group was configured as 32.

### 4.2. Evaluation Metrics

The accuracy, precision, recall, and F1 score can be calculated from the data in the confusion matrix and used as evaluation metrics to assess the performance of the deep learning architecture [39]. The calculation formulas and short descriptions of these evaluation metrics are shown in Table 2.

### 4.3. Hyper-Parameter Optimization

The learning rate is a significant hyper-parameter in the model training process, which represents the update rate of model weights [40]. It is difficult to get the objective function to converge to its local minimum in a reasonable amount of time when setting a learning rate that is too large or too small [41]. In this experiment, five sets of learning rates were designed (0.1, 0.01, 0.001, 0.0001, and 0.00001), and the best one was selected from among them by experiment.

When it comes to CNN models, the trained model is prone to overfitting if the model has a lot of parameters and the training sample is small. Dropout could mitigate the risk of overfitting and achieve the effect of regularization to a certain extent, and the activation values of some neurons are allowed to be randomly set to zero during training to reduce the dependency between some neurons, thus suppressing overfitting and improving the model’s generalization capability [42]. The model development process of the experiment used a combination of dropout and learning rate adjustment for the optimal selection of hyper-parameters; four sets of dropout parameters were designed (0, 0.25, 0.5, and 0.75).

#### 4.3.1. Influence of Dropout

The loss, accuracy, precision, recall, and F1 score values of the 20 test groups are shown in Table 3. From Table 3, it is apparent that the combination of learning rate and dropout had a remarkable effect on the loss, accuracy, and F1 score values.

In the test set, when the learning rate was 0.1, 0.01, 0.0001, and 0.00001, the loss value gradually increased with the increase in dropout. When the learning rate was 0.1, 0.01, 0.001, and 0.0001, and dropout was 0, the model did not obtain the highest accuracy rate, which indicates that configuring an appropriate dropout value can effectively suppress the overfitting of the model. When the learning rate was 0.00001, the test set’s accuracy and F1 score value decreased as the dropout rate increased, while the loss value gradually increased, indicating that the model did not produce overfitting.

#### 4.3.2. Influence of Learning Rate

**Figure 5:**
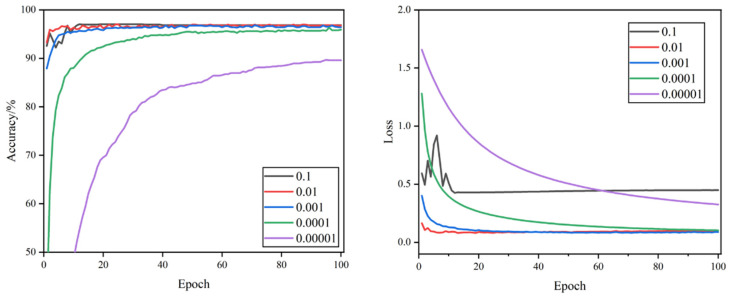
Validation accuracy and loss curves of different learning rates when dropout = 0.

**Figure 6:**
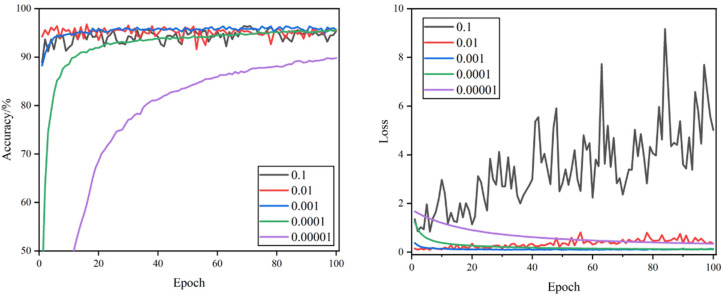
Validation accuracy and loss curves of different learning rates when dropout = 0.25.

**Figure 7:**
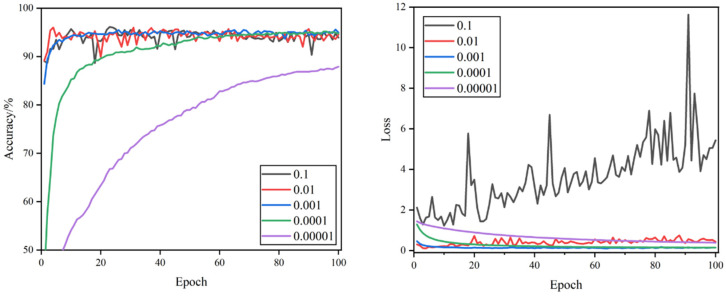
Validation accuracy and loss curves of different learning rates when dropout = 0.5.

**Figure 8:**
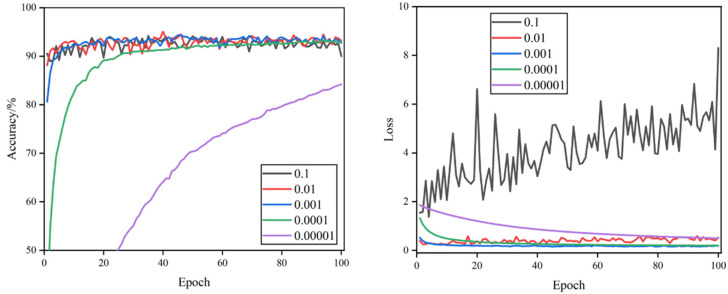
Validation accuracy and loss curves of different learning rates when dropout = 0.75.

The curves of validation accuracy and validation loss during the training process are shown in Figure 5, Figure 6, Figure 7 and Figure 8. By comparing the curves corresponding to the learning rates of the five sets, it can be seen that the rate of curve convergence to smoothness increased with the learning rate. The loss values were close to 0 when the learning rate was 0.001 and 0.0001.

When the learning rate was 0.1, the validation accuracy and validation loss value fluctuated significantly. The validation accuracy curve was more undulating, the validation loss value curve lacked a clear convergence trend, and large loss values occurred at times. When the learning rate was 0.01, the validation loss value curve had a clear convergence trend and was more stable, and the fluctuation of the validation accuracy curve was smaller, but there were still significant fluctuations that required model parameter optimization. When the learning rate was 0.001, the convergence speed of validation accuracy and validation loss was the fastest, the curve of validation accuracy was relatively smooth, the curve of the validation loss value was the smoothest, and the loss value was closest to 0, which indicates that the model has a strong fitting ability. When the learning rate was set to 0.0001, the validation accuracy and validation loss value curves converged steadily, though the convergence speed was relatively slow and the loss value was slightly higher. When the learning rate was 0.00001, the convergence of the validation accuracy and validation loss value curves was the slowest, and neither completely converged, proving that the learning rate was set too small, and it would take a long time to search the optimal parameters.

Combined with Table 3, comparing 20 experimental hyper-parameter combinations, the highest test accuracy rate of 98.85%, F1 score value of 98.87%, and the minimum loss value of 0.033 were achieved when the learning rate was 0.001 and dropout was 0.5. It was the best-performing hyper-parameter combination compared to other combinations.

### 4.4. Frozen Layer Optimization

Transfer learning is a method that aims at applying knowledge learned in one domain to a similar domain [43,44,45]. Through transfer learning, untrained models can acquire the parameters of trained models, thus improving learning efficiency and achieving better accuracy [46].

In this experiment, the training parameters of MobileNet were downloaded from ImageNet for the detection of surface defects in fresh-cut cauliflower. The first few layers of the deep learning model learn the most common patterns of the image, which are relatively easy to migrate. As the convolutional layers in the CNN become deeper and deeper, the content learned becomes more and more specific, the learned parameters may not be fully applicable to the detection of fresh-cut cauliflower surface defects, and negative migration may even occur. Therefore checking if there is negative migration and how to avoid it is the problem to be solved.

To transfer the pre-trained MobileNet model to the task of fresh-cut cauliflower surface defect detection and classification, the pre-trained MobileNet model was reconstructed in the structure of a “feature extraction module + classifier module”, as shown in Table 4. From layer 1 to 86, the feature extraction module was used for feature extraction such as color and texture. From layers 87 to 89, the classifier module was used for feature dimensionality reduction and outputting category labels.

To select the optimal frozen layer, different numbers of depth-wise separable convolution modules were frozen sequentially according to the structure of MobileNet, i.e., different numbers of layers were selected to be frozen. For example, when FL = 10, only 1~10 layers were frozen, and when the frozen layer = 86, the whole feature extraction module was frozen. The model evaluation metrics are shown in Table 5.

According to Table 5, the best performance of the model was achieved when the frozen layer = 80. The results indicate that the original parameters directly migrated from the MobileNet model cannot be directly used for the detection and recognition of fresh-cut cauliflower surface defects, and the existing fresh-cut cauliflower image samples must be used to further adjust the original parameters of MobileNet to obtain the best recognition results.

In this experiment, a CNN combined with transfer learning was used to detect surface defects of fresh-cut cauliflower, and MobileNet was used for training 20 combinations of the hyper-parameter to acquire the best training results, and the best number of the frozen layer was determined by freezing different layers of convolutional layers in the model. In this experiment, the model obtained the best results when the dropout was 0.5, the learning rate was 0.001, and the frozen layer was 80.

### 4.5. Comparative Analysis of Models

To attest to the prominent performance of MobileNet, after optimizations, we applied MobileNet and the other three models (VGG19, InceptionV3, and NASNetMobile) in fresh-cut cauliflower surface defect detection. These three models have successfully demonstrated their excellent performance through competition and application by researchers, so they were used as comparison experiments in this research. Comparison results are shown in Table 6. MobileNet had a significant advantage in test set loss, accuracy, and F1 score values. We can notice from the comparison that MobileNet was more appropriate for the detection of fresh-cut cauliflower surface defects.

**Figure 9:**
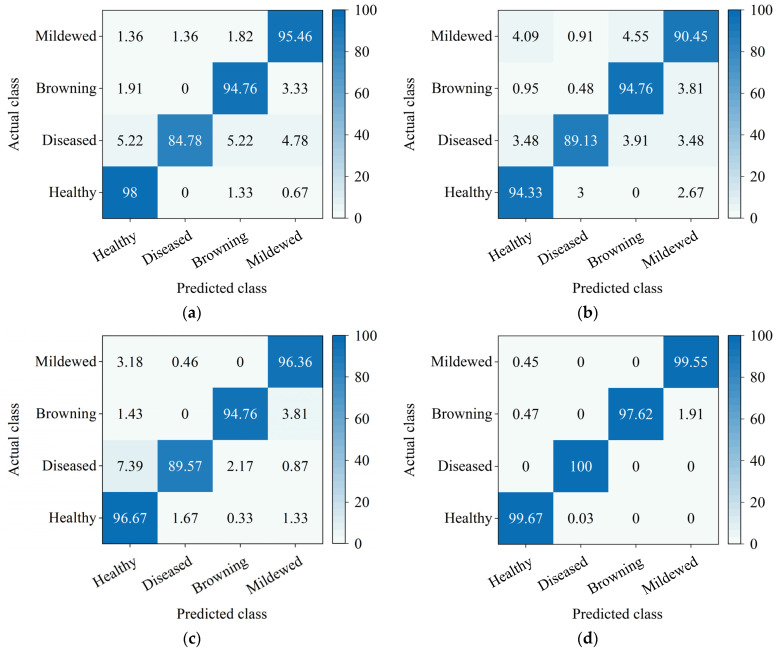
Confusion matrices of four models: (**a**) VGG19; (**b**) InceptionV3; (**c**) NASNetMobile; (**d**) MobileNet. Correct classification is represented by the saturated cells on the diagonal. Off-diagonal cells indicate incorrect classification. Each cell displays the percentage of total classifications.

A confusion matrix is a visualization tool that shows the results of its predictions for a classification task, especially for supervised learning, and in unsupervised learning is generally called a matching matrix. It uses percentages to summarize the ratio of correct and incorrect predictions and breaks them down by each category. The confusion matrix shows which part of the classification model makes errors when making predictions, allowing us to know not only the errors made by the classification model but, more importantly, the type of errors that occurred. The confusion matrices of the four models are shown in Figure 9.

It can be seen from Figure 9 that diseased cauliflower was the most susceptible to error and was misclassified as other categories in VGG19, InceptionV3, and NASNetMobile. Because the color characteristics are not obvious in some samples of diseased cauliflower, they caused misidentification. The improved MobileNet model achieved great classification performance in this experiment, as well as good feature extraction capability.

### 4.6. Visualization of CNN

**Figure 10:**
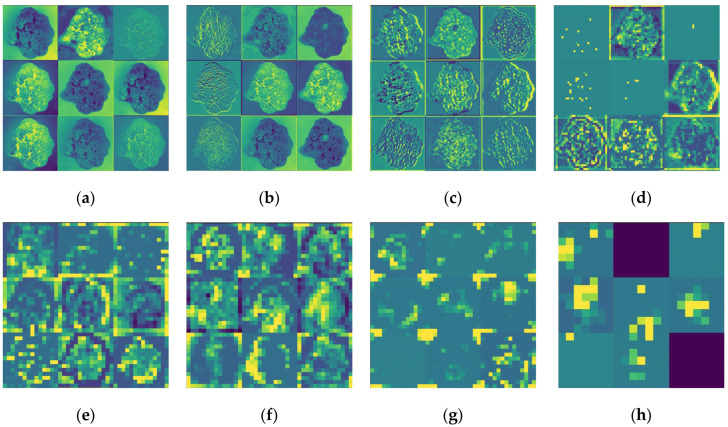
Feature visualization of the MobileNet model: (**a**) convolution; (**b**) depth-wise separable convolution_1; (**c**) depth-wise separable convolution_3; (**d**) depth-wise separable convolution_5; (**e**) depth-wise separable convolution_7; (**f**) depth-wise separable convolution_9; (**g**) depth-wise separable convolution_11; (**h**) depth-wise separable convolution_13.

Deep learning is often referred to as a “black box”, where the inner algorithm cannot be observed, but the representation learned by the CNN can be visualized [47]. Convolutional layer feature visualization can help explain how the CNN transforms the input and shows the features learned by the convolutional layers to provide more insight into the process of the CNN analysis of fresh-cut cauliflower surface defect features. During the training stage, we presented some visualization results to obtain a better explanation of how the model works. Feature maps of fresh-cut cauliflower are shown in Figure 10.

In Figure 10, we show the feature maps of eight different depths of the CNN. In Figure 10a, the feature map is closer to the input image, and as the number of layers increases, the feature map is closer to the output of the model, which becomes more and more abstract and not humanly interpretable.

Deep neural networks can effectively act as an information distillation pipeline (IDP), inputting the original data and repeatedly transforming it to filter out irrelevant information and amplifying and refining the useful information. From the above results, it can be seen that as the layers of the convolutional neural network deepen, the characteristics extracted by the convolutional layer become more and more abstract, with less and less visual information about the original fresh-cut cauliflower images and more and more information about the target category. Therefore, the proposed method in this paper can effectively obtain the surface information of fresh-cut cauliflower.

## 5. Discussion

In this experiment, the CNN combined with transfer learning was used for detecting surface defects of fresh-cut cauliflower. Twenty combinations of hyper-parameters were used to determine the best hyper-parameters for model training, and then different layers of the CNN were frozen to determine the best number of frozen layers with the help of the transfer learning method. The experimental results showed that the model achieved the best results in the test when the learning rate was 0.001, the dropout was 0.5, and the number of frozen layers was 80.

In the MobileNet network applied to the quality detection of agricultural products, Ashwinkumar S et al. proposed a model for the identification of plant leaf diseases named OMNCNN. This model operated in different stages, where the MobileNet model was used as the base model in the feature extraction stage. Experimental results showed that OMNCNN achieved excellent performance, with the F1 score reaching 0.985 [48]. To implement plant disease leaf detection on cell phones, Liu Yang et al. performed transfer learning on two lightweight CNNs, MobileNet and InceptionV3, to obtain two crop disease classification models, and ported them to Android cell phones, respectively. Although the overall recognition accuracy of InceptionV3 was slightly higher on the test set, the recognition balance and model size of MobileNet was more advantageous. When both models were ported to the mobile phone side, MobileNet occupied less memory and recognized images faster, indicating that MobileNet is more suitable for plant disease recognition applications on the cell phone side [49]. Aditya Rajbongshi et al. proposed a method for detecting rose diseases with MobileNet. They performed a comparative analysis by using transfer learning and not using transfer learning. By comparison, the MobileNetV1 model with transfer learning achieved better experimental results, reaching a 95.63% accuracy rate [50].

The results of the above research show that it is feasible to use MobileNet for fresh-cut cauliflower surface defect detection. A CNN has the advantage of a strong self-learning capability in image processing; in this respect, it is fundamentally different from traditional machine learning. A CNN obtains hierarchical feature representation by abstracting and analyzing the original input data and automatically learning layer-by-layer transformation, and finally obtains quantities of data features, and this method is more conducive to classification as well as feature visualization. For the future commercialization and market development of fresh-cut cauliflower, combining CNN with transfer learning may be a solution for the automatic detection and classification of fresh-cut cauliflower surface defects, which can improve detection efficiency and reduce production costs.

## 6. Conclusions

This research proposed a fresh-cut cauliflower surface defect recognition model to achieve the automatic recognition of fresh-cut cauliflower in quality inspection work. Model building was based on a CNN and transfer learning, and the model optimization was achieved by selecting the optimal combination of training hyper-parameters, and by adjusting the different number of frozen layers; the parameters downloaded from ImageNet were optimally integrated with the parameters trained by our experiment. Finally, we compared the improved MobileNet with VGG19, InceptionV3, and NASNetMobile, and the conclusions we have drawn are:

1. By comparing 20 combinations of dropout and learning rates, we found that using a suitable combination of hyper-parameters can improve the model’s generalization performance and accuracy while ensuring training stability. Therefore, the optimal combination of hyper-parameters (dropout = 0.5, learning rate = 0.001) could obtain the highest test accuracy of 98.85% and F1 score of 98.87%.
2. The MobileNet model used the transfer learning method by freezing the different numbers of CNN layers, and when the number of frozen layers was 80, the accuracy improved by 0.42%, the F1 score improved by 0.37%, and the model loss value reduced by 0.008.
3. The improved MobileNet model was compared with VGG19, InceptionV3, and NASNetMobile on the fresh-cut cauliflower test set. By comparing the test results, the improved MobileNet model has the best performance. Therefore, MobileNet was more suitable for fresh-cut cauliflower surface defect detection.

For this research, we improved the accuracy and F1 score of the model by adjusting only the training hyper-parameters and combining ImageNet with our trained parameters, but the variety of datasets tested was small, so limitations still exist. In future research, we have a schedule to develop a more comprehensive dataset of fresh-cut cauliflower surface defects and build a more streamlined model by minimizing the network parameters while maintaining a high accuracy rate. In addition, we plan to deploy the model to mobile devices while maintaining its stability and accuracy, allowing this model to be more widely used so that it is more conducive to the industrialization and commercialization of fresh-cut cauliflower.

## Figures and Tables

**Table 1:** The dataset descriptions.

Classes	Original Dataset	Augmented Dataset	Training Set	Validation Set	Test Set
Healthy	150	1500	900	300	300
Diseased	103	1030	610	210	210
Browning	114	1140	700	220	220
Mildewed	112	1120	670	220	230
Total	479	4790	2880	950	960

**Table 2:** Evaluation metrics.

Evaluation Metric	Calculation Formula	Short Description
Accuracy	$\mathrm{Accuracy}\left(\%\right)=\frac{\mathrm{TP}+\mathrm{TN}}{\mathrm{TP}+\mathrm{FP}+\mathrm{FN}+\mathrm{TN}}\;\times \;100$	The ratio of all predictions is correct (including positive and negative categories) to the total number of samples.
Precision	$\mathrm{Precision}\left(\%\right)=\frac{\mathrm{TP}}{\mathrm{TP}+\mathrm{FP}}\;\times \;100$	The ratio of correct predictions of positive categories to samples predicted to be positive.
Recall	$\mathrm{Recall}\left(\%\right)=\frac{\mathrm{TP}}{\mathrm{TP}+\mathrm{FN}}\;\times \;100$	The ratio of correct predictions of positive categories to all actual positive samples.
F1 score	$F1-\mathrm{score}\left(\%\right)=2\;\times \;\frac{\mathrm{Precision}\;\times \;\mathrm{Recall}}{\mathrm{Precision}+\mathrm{Recall}}\;\times \;100$	The harmonic mean of precision and recall.

Note: TP means the number of predictions of positive categories to positive categories; TN means the number of predictions of negative categories to negative categories; FP means the number of predictions of negative categories to positive categories; FN means the number of predictions of positive categories to negative categories.

**Table 3:** Results of experiments based on MobileNet.

Experiment Code	Learning Rate	Dropout	Loss	Accuracy (%)	Precision (%)	Recall (%)	F1-Score (%)
1	0.1	0	0.2312	97.71	97.83	97.59	97.71
2		0.25	1.4238	97.92	98.01	97.76	97.89
3		0.5	1.6862	97.60	97.95	97.46	97.70
4		0.75	2.8199	95.42	96.38	95.08	95.73
5	0.01	0	0.0467	98.33	98.46	98.25	98.35
6		0.25	0.0736	98.75	98.78	98.72	98.75
7		0.5	0.0912	97.71	97.90	97.59	97.75
8		0.75	0.1913	97.40	97.83	97.22	97.52
9	**0.001**	0	0.0402	98.54	98.62	98.46	98.54
10		0.25	0.0469	98.33	98.47	98.19	98.33
11		**0.5**	0.0410	**98.85**	98.90	98.83	**98.87**
12		0.75	0.0785	97.50	97.85	97.33	97.59
13	0.0001	0	0.0579	98.44	98.50	98.36	98.43
14		0.25	0.0582	98.65	98.81	98.64	98.72
15		0.5	0.0760	98.02	98.24	97.94	98.09
16		0.75	0.1156	98.02	98.19	97.93	98.06
17	0.00001	0	0.2466	95.52	95.87	95.43	95.65
18		0.25	0.2647	94.90	95.44	94.68	95.06
19		0.5	0.2925	94.38	94.72	94.13	94.42
20		0.75	0.3947	90.42	91.43	90.22	90.82

**Table 4:** Structure of the MobileNet model.

Module	Layer	Name	Output Shape
Feature Extraction	1	Input Layer	(None, 224, 224, 3)
	2	Convolution	(None, 112, 112, 32)
	3	Batch Normalization	(None, 112, 112, 32)
	4	ReLU	(None, 112, 112, 32)
	5~10	Depth-wise Separable Convolution_1	(None, 112, 112, 64)
	11	ZeroPadding2D	(None, 113, 113, 64)
	12~17	Depth-wise Separable Convolution_2	(None, 56, 56, 128)
	18~23	Depth-wise Separable Convolution_3	(None, 56, 56, 128)
	24	ZeroPadding2D	(None, 57, 57, 128)
	25~30	Depth-wise Separable Convolution_4	(None, 28, 28, 256)
	31~36	Depth-wise Separable Convolution_5	(None, 28, 28, 256)
	37	ZeroPadding2D	(None, 29, 29, 256)
	38~43	Depth-wise Separable Convolution_6	(None, 14, 14, 512)
	44~49	Depth-wise Separable Convolution_7	(None, 14, 14, 512)
	50~55	Depth-wise Separable Convolution_8	(None, 14, 14, 512)
	56~61	Depth-wise Separable Convolution_9	(None, 14, 14, 512)
	62~67	Depth-wise Separable Convolution_10	(None, 14, 14, 512)
	68~73	Depth-wise Separable Convolution_11	(None, 14, 14, 512)
	74	ZeroPadding2D	(None, 15, 15, 512)
	75~80	Depth-wise Separable Convolution_12	(None, 7, 7, 1024)
	81~86	Depth-wise Separable Convolution_13	(None, 7, 7, 1024)
Classifier	87	GlobalAveragePooling2D	(None, 1024)
	88	Fully Connected Layer	(None, 1, 1, 1024)
	89	Softmax	(None, 4)

**Table 5:** Test results correspond to different frozen layers.

Frozen Layer	Training Parameters	Time (secs)	Loss	Accuracy (%)	Precision (%)	Recall (%)	F1 Score (%)
0	3,211,076	1,689	0.1239	98.44	98.39	98.28	98.34
4	3,210,148	1,632	0.0411	98.96	98.93	98.81	98.87
10	3,207,620	1,381	0.0869	98.44	98.34	98.38	98.36
17	3,198,468	1,224	0.0597	98.85	98.83	98.70	98.76
23	3,180,420	1,000	0.0306	98.75	98.63	98.66	98.65
29	3,145,732	1,021	0.0619	98.54	98.56	98.48	98.52
36	3,076,868	979	0.0394	98.65	98.62	98.61	98.62
43	2,941,956	930	0.0252	99.06	99.06	99.05	99.06
49	2,673,156	909	0.0514	99.06	99.00	99.01	99.00
55	2,404,356	954	0.0491	98.96	98.93	98.82	98.88
61	2,135,556	894	0.0849	97.60	97.84	97.37	97.60
67	1,866,756	896	0.0976	98.54	98.62	98.38	98.51
73	1,597,956	849	0.0592	99.17	99.28	99.11	99.20
**80**	1,065,988	885	0.0330	**99.27**	99.26	99.21	**99.24**
86	4,100	806	0.0465	98.33	98.49	98.27	98.38

**Table 6:** Performance comparison of different classification models.

Model	Memory (MB)	Loss	Accuracy (%)	Precision (%)	Recall (%)	F1 Score (%)
VGG19	549	0.2056	93.54	93.65	93.25	93.45
InceptionV3	92	0.2393	92.29	92.18	92.17	92.17
NASNetMobile	23	0.2060	94.48	94.88	94.34	94.61
MobileNet	16	0.0330	99.27	99.26	99.21	99.24

## Data Availability

Data is contained within the article.
